# Comparative evaluation of mineral profiles in different blood specimens of dairy cows at different production phases

**DOI:** 10.3389/fvets.2022.905249

**Published:** 2022-10-18

**Authors:** Hussein Awad Hussein, Anja-Elivera Müller, Rudolf Staufenbiel

**Affiliations:** ^1^Internal Veterinary Medicine, Department of Animal Medicine, Faculty of Veterinary Medicine, Assiut University, Assiut, Egypt; ^2^Vet Med Labor GmbH, IDEXX Laboratories, Kornwestheim, Germany; ^3^Klinik Für Klauentiere der Freien Universität Berlin, Berlin, Germany

**Keywords:** anticoagulants, dairy cows, minerals, sample types, trace elements, variations

## Abstract

**Background:**

Evaluation of mineral profiles including essential and toxic elements in dairy cows provides fundamental information for bovine practitioners during regular herd supervision and monitoring. The present research was designed to investigate the variations of mineral profiles in different blood specimens of dairy cows at different lactation stages.

**Methods:**

This study was divided into two parts: the first included 32 cows, which were classified into four groups according to their lactation stages, and the second involved 10 cows at mid-lactation. The concentrations of copper (Cu), zinc (Zn), selenium (Se), manganese (Mn), barium (Ba), strontium (Sr), calcium (Ca), magnesium (Mg), total phosphorous (P), sulfur (S), cobalt (Co), silicon (Si), lithium (Li), nickel (Ni), thallium (Tl), boron (B), aluminum (Al), uranium (U), and arsenic (As) were measured in serum, ethylene diamine tetraacetic acid (EDTA) plasma, heparin plasma, and EDTA whole blood samples.

**Results:**

The concentrations of Cu, Zn, Fe, Mn, Ba, and Sr showed significant variations among the dairy cows of different lactation stages (*p* < 0.05). Strong regressions were determined between the mineral concentrations in individual and pooled samples (*R*^2^ = 0.991, *p* = 0.000). In comparison to other blood sample types, the concentration of Cu, Ba, and Sr was higher in EDTA plasma (*p* < 0.000). In addition, the values of Zn, Se, Fe, and Mn were significantly increased in heparin and EDTA whole blood samples. Concentrations of Ca and Mg, and P were higher in EDTA plasma, and EDTA whole blood samples, respectively. Furthermore, the mean values of Si, Li, Ni, and Tl showed significant increases in EDTA plasma, while S values were higher in EDTA whole blood samples (*p* < 0.000). Concentrations of Al and U exhibited significant increases in serum samples (*p* < 0.000).

**Conclusion:**

Concentrations of Cu, Zn, Fe, Mn, Ba, and Sr undergo physiological variations among dairy cows at different lactation stages. Therefore, caution should be taken during assessment of these minerals. The concentrations of essential and toxic elements, as well as Ca, P, Mg, and S, varied among the different blood sample specimens, indicating their interpretations should be based on this regard. During dairy herd supervision, the use of pool sample, instead of individual ones, for determination of mineral status may be promising to minimize the costs of individual sample measurements. In general, EDTA plasma may be more suitable for measurements of Ca, Mg, P, and S. It seems that EDTA plasma and heparinized plasma are suited for the estimation of Se and Fe, respectively.

## Introduction

During herd management of dairy cows, it is very important to improve milk production and minimize adverse effects on health, welfare, and reproductive performance. Of the 103 elements in the periodic table, about 30 are currently considered essential or important for the normal health and growth of animals (1). Minerals are essential for the normal functioning of almost all biochemical processes in the animal body (2). The mineral elements are essential components of many enzyme systems. Simple or conditioned deficiencies of mineral elements therefore have profound effects on metabolism and tissue structure in domestic animals (3).

Deficiencies of major and minor minerals are frequent worldwide and are closely associated with poor animal health, production, and reproduction (2). However, excessive intake of major, minor, or accidental minerals also has deleterious effects on health, production, and reproduction of animals (4). Furthermore, the interactions between trace elements are frequently involved in the pathogenesis of numerous nutritional disorders in ruminants such as formation of thiomolybdate complex as a result of the interaction between copper, sulfur, and molybdenum (5), as well as the intestinal competition of zinc with copper, iron, lead, calcium, and cadmium may accentuate nutritional deficiencies or toxicities from these environmental metals (6).

Different biological samples could be used for determination of mineral concentrations. The most common specimen is serum (1); however, plasma samples collected either with lithium heparin or ethylene diamine tetraacetic acid (EDTA) were also used (7). In recent study (8), hair, urine, and feces were used as samples for assessment of mineral status in dairy cows. Little is known about using whole blood as a biological sample for assessment of mineral profiles in dairy cows.

From the clinical point of view, evaluation of mineral profiles including essential and toxic elements in dairy cows provides fundamental information for bovine practitioners during herd supervision. To the best of authors' knowledge, there is little information about the effect of production stage of dairy cows on the mineral status, as well as the comparative evaluation of mineral elements in the different biological specimens (serum, plasma, and whole blood) is miserly. Therefore, the objectives of this study were to (1) determine the concentrations of mineral profiles in dairy cows at different production stages and (2) compare the levels of essential and toxic elements in different blood sample types, including serum, heparin plasma, EDTA plasma, and whole blood.

## Materials and methods

### Management and sampling

The present study was carried out on a dairy herd of Holstein–Friesian cows within the framework of regular herd supervisions carried out by the Clinic of Ruminant and Swine of Free University of Berlin. The age and lactations of animals ranged from 4 to 8 years and 2 to 6 lactations, respectively. All the cows were clinically healthy, and any cow that exhibited illness was excluded from the study on the basis of a clinical examination conducted before the collection of blood samples. All dairy cows were kept in a free stall barn and fed a diet of grass and corn silage and concentrate as totally mixed ration (TMR). Ingredients and chemical composition of the close-up and high-lactation diet are provided (Supplementary material 1). The water supply was available *ad libitum*. Based on the study design and number of animals, the research was divided into two parts, given as follows.

### Part 1

This part included a total number of 32 cows. All cows were classified according to their production stages into four different groups as close-up (3–0 week antepartum, *n* = 8), fresh lactation stage (0–1 week postpartum, *n* = 8), early lactation (3–5 weeks postpartum, *n* = 8), and mid-lactation (15–18 weeks postpartum, *n* =8). From each cow, five blood samples were collected, before the morning main meal, from the coccygeal vein with caution to avoid hemolysis. The first sample was collected in a vacutainer tube without anticoagulant for separation of serum. The second and third samples were collected in vacutainer tubes containing ethylene diamine tetraacetic acid (EDTA) and lithium heparin as anticoagulants for separation of EDTA and heparin plasma, respectively. The fourth and fifth blood samples were kept as whole blood samples in tubes containing lithium heparin and EDTA as anticoagulants, respectively. From each individual sample material, a new pool sample, representing each stage, was prepared to find the relationship between individual and pool samples. To make a pool sample for each blood material (serum, heparin plasma, and heparin whole blood), an equal aliquot (1 ml) from each individual of the same lactation stage (close-up, fresh lactation, early lactation, and mid-lactation) was transferred to a new collecting tube. The total amount of a pool sample was 8 ml as the number of included individuals. The concentrations of copper (Cu), zinc (Zn), selenium (Se), iron (Fe), manganese (Mn), barium (Ba), and strontium (Sr) were measured in individual and pooled blood sample materials, respectively.

### Part 2

A total of 10 different cows, at their mid-lactation (15–18 weeks postpartum), were included in this part. From each cow, four blood samples were harvested with caution from coccygeal veins. All samples were collected just before the morning main feeding (9). The blood samples were mixed with ethylene diamine tetraacetic acid (EDTA) for separation of EDTA plasma, with lithium heparin for separation of heparin plasma, and without anticoagulant for serum separation. The fourth sample was kept as an EDTA whole blood. The concentrations of calcium (Ca), magnesium (Mg), total phosphorus (P), sulfur (S), Cu, Zn, Mn, Fe, cobalt (Co), Se, boron (B), barium (Ba), strontium (Sr), silicon (Si), lithium (Li), nickel (Ni), arsenic (As), aluminum (Al), thallium (Tl), and uranium (U) were determined in all individual blood sample materials.

### Laboratory assays

The analyses of the elements in serum, plasma, and whole blood were carried out by inductively coupled plasma–optical emission spectrometry (ICP-OES) or inductively coupled plasma–mass spectrometry (ICP-MS). The sample preparations and measurements were carried out in accordance with the standard protocols (SOP of IDEXX Laboratories, Kornwestheim, Germany, TO_A001, determination of trace elements in liquids by mean of ICP-OES) using an ICP-OES Vista Pro instrument (Varian/Agilent; Santa Clara, California, United States) for the elements with emission wavelengths as follows: Cu (324.754 nm), Zn (202.548 nm), Mn (257.610 nm), S (181.972 nm), P (185.878 nm), Fe (238.204 nm), Ba (455.403 nm), Sr (407.771 nm), Si (251.611 nm), Ca (315.887 nm), Al (167.019 nm), B (249.772 nm), and Mg (279.553 nm). The emission wavelengths of the elements were free of interferences. The sample preparations and measurements of the ICP-MS method were carried out according to the standard work instructions of SOP IDEXX Laboratories, Kornwestheim, Germany, TO_ A004: determination of trace elements in liquids and aqueous solutions using ICP-MS (Varian 820, Aurora Bruker M90, Plasmaquant MS Analytik Jena, Jena, Germany). The ICP-MS method was used for analyzing the elements (isotopes) of Se (78), Co (59), Ni (60), As (75), Li (7), Tl (205), and U (236). The isotope masses were free of interferences because helium was used as collision gas to avoid atomic clusters in the interface of ICP-MS. The ICP-OES and ICP methods are validated and accredited by IDEXX laboratory, including the coefficient of variations (Supplementary material 2), limit of detection (LOD), and limit of quantification (LOQ; Supplementary material 3).

### Statistical analysis

Data were statistically analyzed using SPSS software (IBM SPSS^®^ analytical program for Windows, version 25). For statistical evaluation, linear mixed procedures were used in both study parts. In the first part, the animal number was the subject and the element item was chosen as a dependent variable. Furthermore, animal groups and sample materials were set as fixed effects, while animal number was the random effect. The model included terms for animal groups, type of samples, group × sample interaction, and animal numbers. The following linear mixed model was used: *Y*_*kjf*_ = μ + *G*_*k*_ + *S*_*j*_ + *GS*_*jk*_ + *N*_*f*_ + ε_*kjf*_

where *Y* is the observed mineral concentration, μ is the overall mean, *Gk* is the fixed effect of animal groups (*k* = 1, 2, 3, 4), *S*_*j*_ is the fixed effect of the sample type (*j* = 1, 2, 3, 4, 5), *GS*_*jk*_ denotes group k–sample j interaction, N*f* is the random effect of animal numbers (*f* = 1, 2... 32), and ε_kjf_ is the residual error.

To find the relationship between individual and pool samples for each mineral, linear regression was carried out, and R square and regression coefficients were estimated.

In the second part of the study, the animal number was set as a subject and the mineral element was set as a dependent variable. In addition, the sample type and animal number were chosen as a fixed and random effects, respectively. The following linear mixed model was used: *Y*_*jf*_ = μ + *S*_*j*_ + *N*_*f*_ + ε_*jf*_

where *Y* is the observed mineral concentration, μ is the overall mean, *S*_*j*_ is the fixed effect of the sample type (*j* = 1, 2, 3, 4), N*f* is the random effect of animal numbers (*f* = 1, 2… 10), and ε_jf_ is the residual error.

In both study parts, the main effects were tested using least square differences (LSD). Effects were considered significant at *P* < 0.05. For each mineral concentration, the data presented as estimated marginal means, standard error of mean (SEM), and 95% confidence intervals. The normal distribution of the residuals was tested using the Shapiro–Wilk test. The residuals of all mineral concentrations were normally distributed, except iron; therefore, the data of iron were arithmetically transformed using natural logs (Ln).

## Results

Table 1 summarizes the results of the linear mixed model of essential and accidental elements in dairy cows at different production stages. Independent of blood sample type, the concentrations of Cu and Zn were significantly increased in fresh lactation and close-up groups, respectively. Selenium levels showed insignificant changes among the cow groups (*P* > 0.05), while Fe and Mn values were lower in close-up and fresh lactation than in late lactation cows. The concentrations of Ba and Sr showed a significant increase in fresh and early-lactation groups. Regardless of the stage of lactation, the concentrations of trace elements showed significant variations among the different blood sample types (Table 2). The concentrations of Cu were lower in serum samples than in other sample types. Furthermore, the concentrations of Zn, Se, Fe, and Mn were significantly increased in heparin and EDTA whole blood samples. In addition, Ba and Sr levels were higher in EDTA plasma than in other blood sample types.

**Table 1:** Summary of the linear mixed model analysis for essential and accidental trace element concentrations in dairy cows at different production phases (*n* = 32).

**Parameters**	**Lactation phases**				***P*-value**	**Estimated marginal means**		
	**Close-up**	**Fresh-lactation**	**Early-lactation**	**Mid-lactation**		**Mean**	**SEM**	**95% confidence interval**	
	**(3–0 WAP)**	**(0–1 WPP)**	**(3–5 WPP)**	**(15–18 WPP)**				
								**Lower bound**	**Upper bound**
Copper (μg/L)	692^b^	849^a^	698^b^	713^b^	0.02	749	19.5	709	789
Zinc (μg/L)	1,635^a^	1,423^b^	1,269^c^	1,456^b^	0.001	1,445	27.8	1,388	1,501
Selenium (μg/L)	187	177	172	186	0.293	181	3.3	174	188
Ln Iron (μg/L)	9.228^b^	9.299^b^	9.348^ab^	9.421^a^	0.014	9.335	0.023	9.288	9.381
Iron^*^ (μg/L)	10,178^b^	10,927^b^	11,476^ab^	12,345^a^		11,328		10,808	11,861
Manganese (μg/L)	3.5^b^	3.6^b^	4.6^a^	4.3^a^	0.007	4.1	0.11	3.8	4.3
Barium (μg/L)	14^b^	24^a^	20^a^	15^b^	0.000	19	0.7	17	20
Strontium (μg/L)	68^b^	90^a^	90^a^	67^b^	0.012	80	3.1	74	86

WAP, week antepartum; WPP, week postpartum.

* Retransformed values.

abc Values with different superscript letters differ significantly P < 0.05.

**Table 2:** Summary of the linear mixed model analysis for essential and accidental trace element concentrations in different blood specimens of dairy cows (*n* = 32).

**Parameters**	**Specimens**					***P*-value**	**Normality of residuals**
	**Serum**	**Heparinized**	**EDTA**	**Heparinized**	**EDTA**		**Shapiro-Wilk**
		**plasma**	**plasma**	**whole blood**	**whole blood**		**Test**
Copper (μg/L)	606^d^	802^b^	881^a^	750^c^	797^bc^	0.000	0.25
Zinc (μg/L)	873^b^	910^b^	900^b^	2,287^a^	2,517^a^	0.000	0.44
Selenium (μg/L)	109^d^	125^c^	129^bc^	283^a^	283^a^	0.000	0.24
Ln Iron (μg/L)	7.2^b^	7.3^b^	7.2^b^	12.5^a^	12.7^a^	0.000	0.23
Iron* (μg/L)	1,407^b^	1,425^b^	1,383^b^	248,646^a^	321,190^a^		
Manganese (μg/L)	1.9^d^	2.5^c^	3.1^b^	6.4^a^	6.9^a^	0.000	0.07
Barium (μg/L)	16^d^	19^c^	25^a^	13^e^	23^b^	0.000	0.28
Strontium (μg/L)	83^b^	89^a^	91^a^	64^d^	76^c^	0.000	0.34

abcd Values with different superscript letters differ significantly P < 0.05. The * symbol means “Retransformed values”.

**Figure 1:**
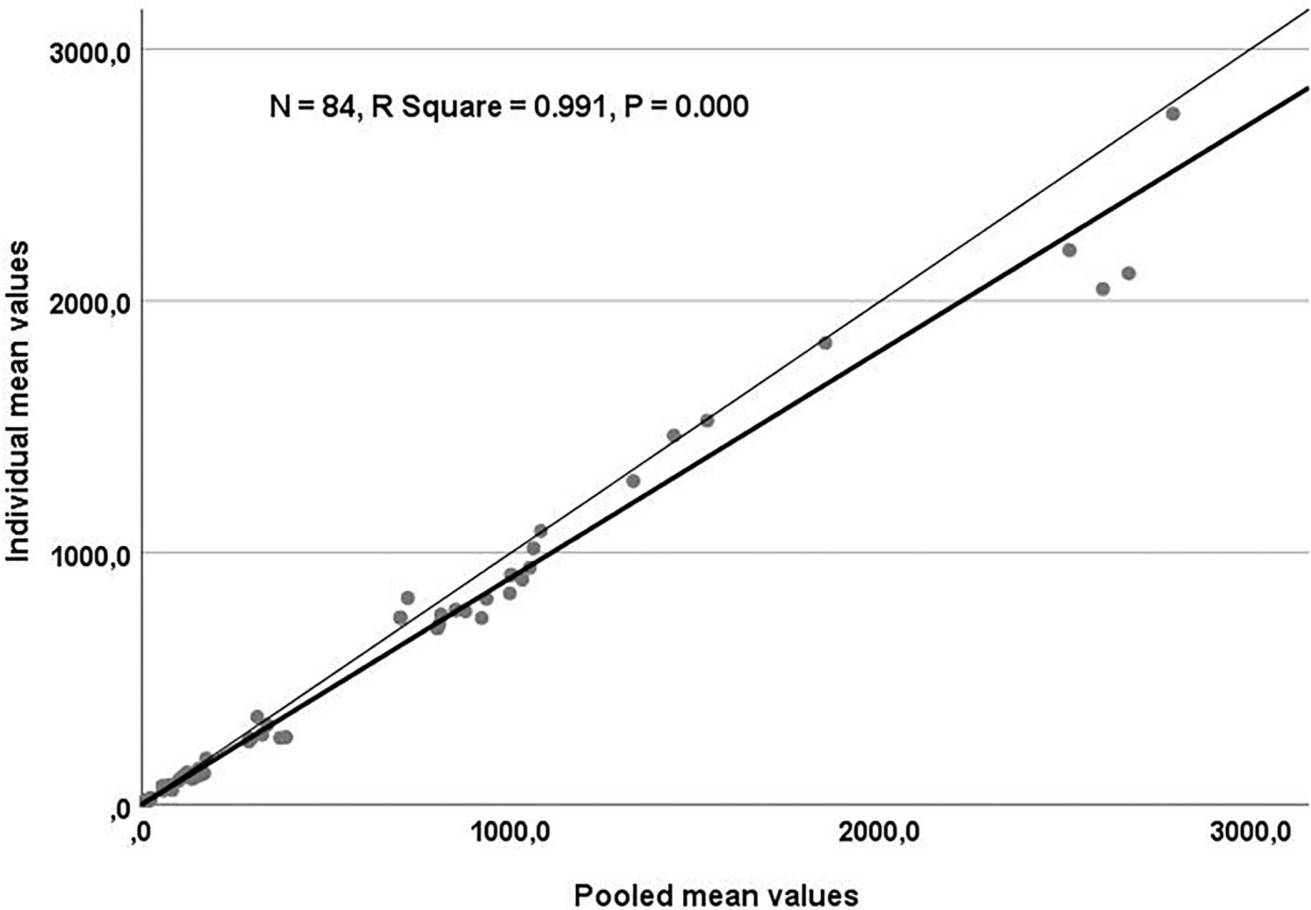
Scatter plot for the regression analysis of essential trace elements. Values obtained in individual and pool samples were plotted and adjusted to a regression line (thick line). The thin line represents the identity line (Y = X).

Table 3 shows the result of linear regression analysis for the trace element concentrations in the individual and pooled samples. A regression coefficient of 0.93 was obtained, relating Cu concentrations in the individual and pool samples (*P* < 0.0001). A significant comparable relationship with a regression coefficient of 0.78 was seen between individual Se and pool Se values (*P* < 0.0001). Individual Sr and pool Sr concentrations were linearly and significantly related, with a regression coefficient of 0.96 (*P* < 0.001). For the overall data of essential trace elements, the relationship between the individual and pool samples is illustrated in Figure 1.

**Table 3:** Linear regression analysis for the essential and accidental trace elements in individual and pooled samples.

**Parameters**	**Regression analysis results**		
	*R* ^2^	***P*** **-value of regression**	**Coefficients of regression**
Average individual Cu—pooled Cu	0.992	0.000	0.929
Average individual Zn—pooled Zn	0.992	0.000	0.872
Average individual Se—pooled Se	0.982	0.000	0.782
Average individual Fe—pooled Fe	0.999	0.000	0.990
Average individual Mn—pooled Mn	0.963	0.000	0.933
Average individual Ba—pooled Ba	0.981	0.000	0.948
Average individual Sr—pooled Sr	0.987	0.000	0.961

In the second part of the present study, the concentrations of major elements showed significant variations in the different blood sample types (Table 4). The highest values of Ca and Mg were observed in EDTA plasma samples. By contrast, the concentrations of total phosphorus were higher in EDTA whole blood samples than in other blood sample types (*P* < 0.05). The concentrations of Cu were lower in serum samples. In addition, the mean values of Zn, Fe, and Se were significantly higher in EDTA whole blood samples, while the concentrations of Mn and Co were higher in EDTA plasma and serum samples, respectively (Table 5). Table 6 summarizes the concentrations of accidental trace elements of different blood sample types of dairy cows. The mean values of Ba, Sr, Si, Li, Ni, and Tl were significantly increased in EDTA plasma samples, while the concentrations of B and S were higher in EDTA whole blood. The highest mean levels for Al and U were seen in serum samples.

**Table 4:** Linear mixed model analysis of major element concentrations in different blood specimens of dairy cows (*n* = 10).

**Parameters**	**Specimens**				***P*-value**	**Estimated marginal means**				**Normality of residuals**
	**Serum**	**Heparinized plasma**	**EDTA plasma**	**EDTA whole blood**		**Mean**	**SEM**	**95% confidence interval**		**Shapiro-Wilk Test**
								**Lower bound**	**Upper bound**	
Ca (mmol/L)	2.3^c^	2.5^b^	2.7^a^	1.8^d^	0.000	2.3	0.03	2.3	2.4	0.50
Mg (mmol/L)	0.94^d^	0.99^bc^	1.1^a^	0.97^cd^	0.000	0.99	0.026	0.93	1.05	0.96
P (mmol/L)	3.7^d^	4.1^c^	4.4^b^	6.5^a^	0.000	4.6	0.11	4.4	4.9	0.47
S (mg/L)	927^d^	1,041^c^	1,133^b^	1,332^a^	0.000	1,108	16	1,072	1,145	0.57

abcd Values with different superscript letters differ significantly P < 0.05.

**Table 5:** Linear mixed model of essential trace element in different blood specimens of dairy cows (*n* = 10).

**Parameters**	**Specimens**				***P*-value**	**Estimated marginal means**				**Normality of residuals**
	**Serum**	**Heparinized plasma**	**EDTA plasma**	**EDTA whole blood**		**Mean**	**SEM**	**95% confidence interval**		**Shapiro-wilk test**
								**Lower bound**	**Upper bound**	
Cu (μg/L)	813^d^	1,134^b^	1,204^a^	962^c^	0.000	1,028	31	958	1089	0.48
Zn (μg/L)	736^d^	799^c^	944^b^	2,609^a^	0.000	1,272	24	1,217	1,327	0.15
LnFe (μg/L)	7.2^c^	7.3^b^	7.3^b^	12.7^a^	0.000	8.6	0.015	8.6	8.7	0.07
Fe^*^(μg/L)	1,339^c^	1,522^b^	1,470^b^	344,552^a^		5,670		5,475	5,866	
Se (μg/L)	64^b^	74^c^	77^c^	180^a^	0.000	99	3	92	106	0.86
Mn (μg/L)	1.98^c^	1.87^c^	6.79^a^	2.47^b^	0.000	3.3	0.17	2.88	3.67	0.55
Co (μg/L)	0.56^a^	0.53^ab^	0.52^ab^	0.49^b^	0.028	0.52	0.08	0.34	0.71	0.15

* Retransformed values.

abcd Values with different superscript letters differ significantly P < 0.05.

**Table 6:** Linear mixed model analysis of accidental and toxic elements in different blood specimens of dairy cows (*n* = 10).

**Parameters**	**Specimens**				***P*-value**	**Estimated marginal means**				**Normality of residuals**
	**Serum**	**Heparinized plasma**	**EDTA plasma**	**EDTA whole blood**		**Mean**	**SEM**	**95% confidence interval**		**Shapiro-wilk test**
								**Lower bound**	**Upper bound**	
Ba (μg/L)	21^b^	22^b^	29^a^	18^c^	0.000	22	1.9	18	27	0.07
Sr (μg/L)	95^c^	101^b^	107^a^	71^d^	0.000	93	4.8	83	104	0.06
Si (mg/L)	1.1^c^	1.2^b^	1.3^a^	1.0^d^	0.000	1.1	0.04	1.03	1.23	0.41
Li (μg/L)	6.6^b^	NA	7.9^a^	5.5^c^	0.000	6.7	0.56	5.4	7.9	0.06
Ni (μg/L)	0.9^b^	0.8^b^	1.2^a^	1.1^a^	0.006	1.01	0.04	0.93	1.1	0.16
Tl (μg/L)	0.047^b^	0.055^b^	0.084^a^	0.064^b^	0.003	0.062	0.006	0.048	0.077	0.86
B (μg/L)	151^d^	168^c^	188^b^	508^a^	0.000	254	14	221	286	0.94
Al (μg/L)	24.8^a^	16.8^b^	15.8^b^	13.6^b^	0.000	17.7	1.8	13.7	21.8	0.62
U (μg/L)	0.164^a^	0.021^b^	0.015^b^	0.010^b^	0.000	0.052	0.004	0.044	0.061	0.12
As (μg/L)	0.46	0.38	0.41	0.44	0.129	0.42	0.04	0.34	0.51	0.79

NA, not applicable.

abcd Values with different superscript letters differ significantly P < 0.05.

**Figure 2:**
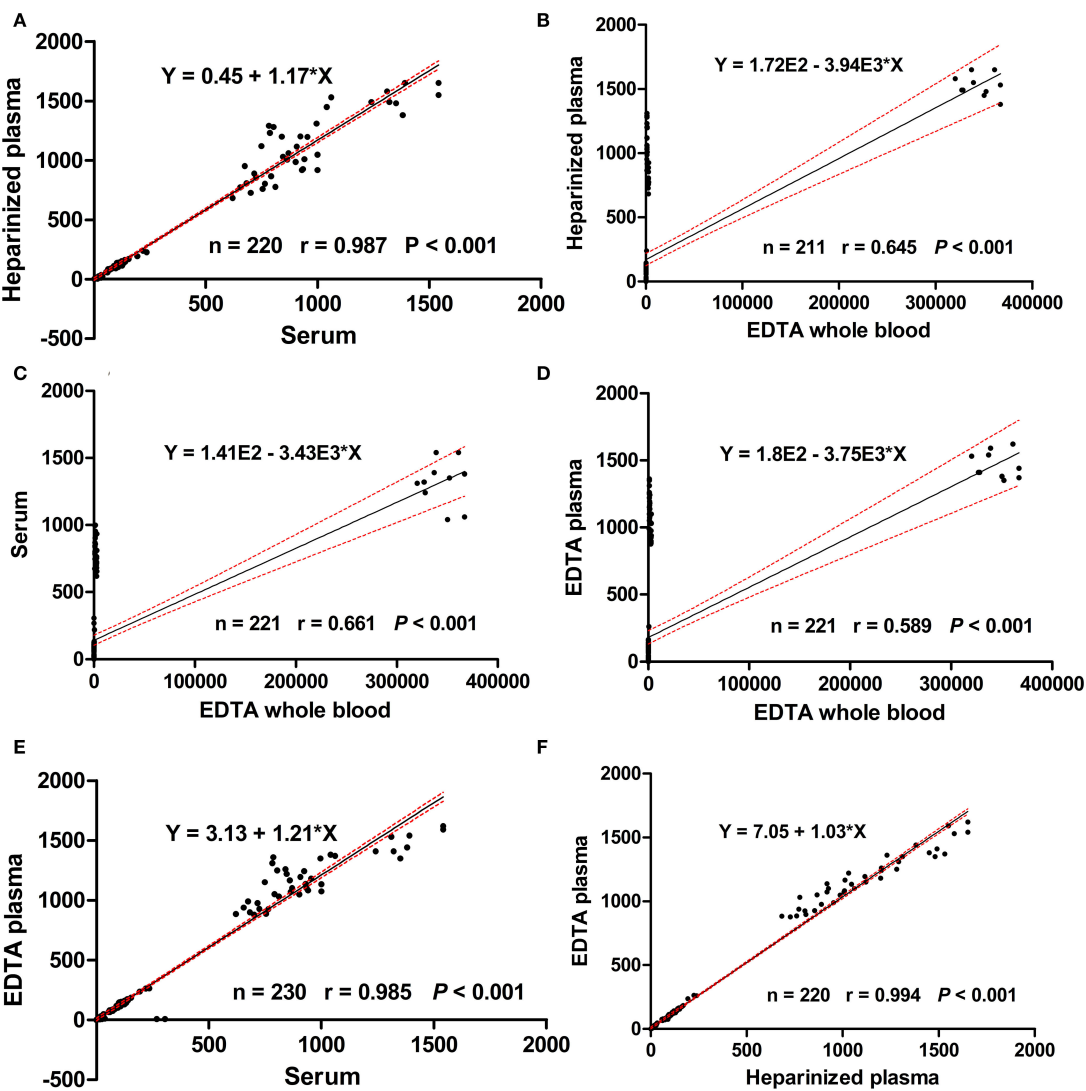
Scatter plots for the regression analysis of mineral concentrations in heparinized plasma vs. serum **(A)**, heparinized plasma vs EDTA whole blood **(B)**, serum vs ETDA whole blood **(C)**, EDTA plasma vs EDTA whole blood **(D)**, EDTA plasma vs serum **(E)**, and EDTA plasma vs heparinized plasma **(F)**. Black lines represent the regression lines, the 2 dashed red lines represent the 95% confidence intervals.

For the major elements, Table 7 lists the relationships among the different blood specimens. For Ca, a strong significant correlation coefficient was noticed between serum and EDTA plasma only (*r* = 0.874, *P* < 0.01). Moreover, a moderate correlation coefficient was observed between heparinized plasma and EDTA plasma for S (*r* = 0.760, *P* < 0.01). Table 8 shows Pearson's correlation coefficients for essential trace elements in different blood specimens. For Se, moderate association was determined between serum and EDTA plasma (*r* = 0.775, *P* < 0.01). For the overall data of mineral concentrations, strong correlation was detected between EDTA plasma and heparinized plasma (*r* = 0.994, *P* < 0.001; Figure 2).

**Table 7:** Pearson's correlation coefficients for major elements in different blood specimens (*n* = 10).

**Parameters**	**Comparative relationships**					
	**Serum vs**.	**Serum vs**.	**Serum vs**	**Heparinized plasma vs**.	**Heparinized plasma vs**.	**EDTA plasma vs**.
	**ETDA blood**	**heparinized plasma**	**EDTA plasma**	**EDTA blood**	**EDTA plasma**	**EDTA blood**
Ca (mmol/L)	0.874^**^	0.076	0.147	0.412	0.608	0.493
P (mmol/L)	0.524	0.674^**^	0.762^**^	0.597	0.902^**^	0.607
Mg (mmol/L)	0.859^**^	0.708^**^	0.852^**^	0.760^**^	0.882^**^	0.770^**^
S (mmol/L)	0.240	−0.036	−0.050	0.405	0.760^**^	0.490

* P < 0.05.

** P < 0.01.

**Table 8:** Pearson's correlation coefficients for essential trace elements in different blood specimens (*n* = 11).

**Parameters**	**Comparative relationships**									
	**Serum vs**.	**Serum vs**.	**Serum vs**.	**Serum vs**.	**Heparinized**	**Heparinized**	**Heparinized**	**EDTA**	**EDTA**	**EDTA**
	**heparinized**	**heparinized**	**EDTA**	**EDTA**	**plasma vs**.	**plasma vs**.	**plasma vs**.	**plasma vs**.	**plasma vs**.	**blood vs**.
	**blood**	**plasma**	**blood**	**plasma**	**heparinized**	**EDTA**	**EDTA**	**heparinized**	**EDTA**	**heparinized**
					**blood**	**blood**	**plasma**	**blood**	**blood**	**blood**
Cu (μmol/L)	0.877^**^	0.799^**^	0.882^**^	0.923^**^	0.673^*^	0.847^**^	0.772^**^	0.926^**^	0.856^**^	0.888^**^
Zn (mmol/L)	0.230	0.896^**^	0.052	0.666^*^	0.284	0.042	0.725^*^	0.266	0.200	0.892^**^
Se (mmol/L)	0.141	0.379	−0.141	0.775^**^	0.487	0.208	0.333	0.166	−0.127	0.642^*^
Fe (mmol/L)	0.208	0.973^**^	0.042	0.127	0.261	0.046	0.237	0.035	−0.090	0.702^*^
Mn (μmol/L)	0.575	0.931^**^	0.208	0.702^**^	0.757^**^	0.390	0.840^**^	0.756^**^	0.463	0.817^**^
Ba (μmol/L)	0.898^**^	0.978^**^	0.507	0.703^*^	0.901^**^	0.521	0.733^*^	0.771^**^	0.184	0.340
Sr (μmol/L)	0.858^**^	0.972^**^	0.890^**^	0.822^**^	0.827^**^	0.819^**^	0.823^**^	0.858^**^	0.668^*^	0.798^**^

* P < 0.05.

** P < 0.01.

## Discussion

Minerals are very essential for animal health, production, and reproduction. Different biological samples could be used for the determination of mineral profiles. The concentrations of minerals may be varied according to the type of biological specimens; therefore, the present study was designed to determine the possible variations in different blood samples, including serum, heparin plasma, EDTA plasma, and whole blood samples at different lactation stages.

The significant increase in Cu in the group of fresh lactation cows suggested that dairy cows around the calving stage experience a physiological acute-phase response as a result of oxidative stress during the transition period (10, 11). In a previous study (12), the authors reported increased Cu immediately after calving of dairy cows. Furthermore, Hussein and Staufenbiel (13) attributed such increase in Cu to increased ceruloplasmin activity, which represents 95% of plasma Cu (14). In the current study, the concentration of Zn showed a significant decrease in the groups of fresh, early, and late lactations in comparison to close-up cows. Such drop may be explained by the stresses of calving and/or milk production. It has been reported that Zn acts as a cofactor for zinc-dependent superoxide dismutase enzyme, which is a part of the primary antioxidant system of all vertebrates (15). In accordance with the present finding, Noaman et al. (16) observed lower Zn concentrations in lactating cows than in those during the dry period.

Iron is a component of cytochromes and Fe-dependent proteins; also, it is a constituent of several Fe-activated enzymes (17, 18). Manganese is an integral part of many enzymes, including arginase, superoxide dismutase, and pyruvate carboxylase (19). In the current research, the lowest values of Fe and Mn were noticed in the group of close-up cows. Such drop could be explained by consumption or withdrawal of these elements by the growing calves during the prenatal stage. It was mentioned elsewhere (20), the fetal liver Fe concentration was higher than the dam cows. In the present research, increased Ba and Sr concentration in fresh and early-lactation cows could be explained by increased feed intake just after calving. This finding was inconsistent with the result of a recent research in dairy cows (21).

In comparison to those in plasma and whole blood, decreased Cu concentrations in serum might be due to trapping of the copper containing protein (ceruloplasmin) in the fibrin clot formation (22). It was reported in a previous study in dairy cows (23) that the serum activity of ceruloplasmin was 30% lower in serum than in heparin plasma. A depletion of Cu in serum up to 50% caused by the clotting process was reported (24). Furthermore, decreased concentrations of Zn in serum may be attributed to its role in thrombosis and coagulation process of the blood. Zinc is considered an important cofactor in the regulation of platelet aggregation and serves as a modulator of hemostasis and thrombosis (25). In comparison to its serum values, increased Se concentrations in plasma and whole blood samples may be due to the higher level of Se in these blood sample types as it was mentioned that in human plasma, Se is associated with three proteins including selenoprotein P (52%), glutathione peroxidase (39%), and albumin (9%) (26). Moreover, whole blood possesses the additional erythrocyte compartment with its own distinct form of glutathione peroxidase (27). Moreover, Luna et al. (28) reported decreased Se levels in serum samples compared with heparin plasma, and they attributed that to loss of Se during the clotting process. In addition, Maas et al. (29) concluded that using serum Se concentration might not be appropriate to assess the nutritional status of Se in cattle. Moreover, concentrations of Se in serum have been used as an index of Se status in dairy cows (30).

In the present research, the highest values of Fe and Mn were noticed in whole blood specimens. Such elevation may be attributed to the high content of Fe and Mn in the red blood cells. It was reported elsewhere (31) that ~25 mg of Fe is taken up by the erythroid progenitor cells in the bone marrow every day to support the daily production of 200 billion new red blood cells. Furthermore, Garnica (32) reported that Mn is an important and essential element for heme and hemoglobin synthesis. In contrast to the present study, it was found that the concentration of Mn was higher in serum samples than in heparin plasma (28). In this study, the concentrations of Ba and Sr were higher in EDTA plasma than in other blood sample types. This finding is in accordance with a previous study (21). Very strong correlations were noticed between trace elements in the individual and pooled samples in the current work, indicating pooled samples can provide group-based diagnostic information for detection of mineral status in dairy herds and could be used as a monitoring tool to minimize the costs of individual samples. In a research study on human serum and whole blood samples (33), the authors demonstrated good agreement for mineral concentrations between the average individual values and that determined in the pooled samples.

The concentrations of Ca and Mg, and P were significantly higher in EDTA plasma samples than in other blood sample types. There is no good explanation for these unexpected results. It is well-known that EDTA is not a suitable anticoagulant for many blood constituents because of its chelating effect (34). The EDTA complex should not be the reason for these unexpected results because the complex will be destroyed by using ICP methods. This is an advantage of these analytical methods compared with other techniques, such as flame photometry, flame atomic absorption, graphite furnace atomic absorption, and cold vapor/hydride generation atomic absorption (35). However, comparable changes in minerals and biochemical analytes induced by EDTA have been described in humans (36), cattle (37), and buffaloes (38). The concentrations of P are highest in EDTA whole blood samples, indicating higher contents of P in red blood cells. As it was mentioned in a previous study (39), inorganic phosphorus is a very important element for intact and integrity of red blood cells. It was reported that low phosphorus intake in cows is contributed to hemolytic anemia and red blood cell metabolic disorders (40). In the current study, the concentration of Co was significantly decreased in EDTA whole blood samples in comparison to other blood sample types. Furthermore, Luna et al. (28) reported no significant changes between Co concentrations in serum and heparin plasma samples.

Accidental elements are naturally occurring in the Earth's crust. However, a rapid increase in industrial and agricultural activities over the last few years has contributed greatly to the distribution of these elements into the environment (41). Accidental elements will accumulate in the animal food chain and subsequently a major route of exposure to dairy cows. Frieden (42) reported that some accidental elements, such as B, Ba, Sr, Si, Li, and As, may play a role in various physiological functions in the human body, but no specific identified biochemical functions. Furthermore, Nielsen (43) listed B, Li, Ni, and Si as occasionally beneficial elements because they can also be regarded as essential, albeit in “ultratrace” concentrations. In addition, Tl, Sr, Tl (44), Al, and As (45) were classified as potentially toxic elements. Increased exposure to trace elements, particularly toxic metals, has caused concern in the livestock population (44).

In the current research, the sample type significantly affected the concentrations of most studied elements, except As, as the concentrations of Ba, Sr, Si, Li, Ni, and Tl were higher in EDTA plasma, and levels of B and S were increased in EDTA whole blood samples. To the best of the authors' knowledge, there are no detailed explanations for such variations, warranting further research in this regard. However, Luna et al. (28) found no significant difference between serum and heparin plasma concentrations of Ba, Sr, Li, Ni, B, and As. In this study, concentrations of Al and U were significantly decreased in heparin and EDTA plasma, and EDTA whole blood samples in comparison to their serum levels. Such difference may be due to fluid exchange between plasma and blood cells. This postulation was supported in previous studies (7, 46), and the authors have attributed that to a fluid shift from erythrocytes into plasma in response to increased osmolality produced by the addition of anticoagulants such as citrate or EDTA. It is well-known that albumin is responsible for the regulation of blood osmotic pressure. In addition, the concentrations of albumin (7, 46) and Al (7) were lower in plasma specimens than in serum samples.

The concentrations of essential and toxic elements get altered among the different studied blood specimens, which could be explained by blood coagulation, type of anticoagulant, and presence of cells. Therefore, the type of sample should be taken in consideration during interpretation of these elements. Higher Ca, Mg, P, and S levels in EDTA plasma with variable relationships among the different specimens were noticed by using the present analytical method, indicating no obvious chelation effect and suggesting the use of EDTA plasma for estimation of these minerals. This explanation was supported in a recent research in human (47); the authors found higher levels of Mg and Ca in EDTA plasma than in heparinized plasma. Although the concentration of Se was higher in EDTA plasma than in serum, moderate relationship was noticed between serum and EDTA plasma Se levels, indicating the superior use of EDTA plasma for measurement of this element. By contrast, Luna et al. (28) reported very strong correlation for Se between serum and plasma. For Fe, although a very strong correlation coefficient was detected between serum and heparinized plasma, its concentration was lower in serum than in heparinized plasma, recommending the use of heparinized plasma for determination of this element. However, the concentration of iron was significantly higher in serum than in plasma (48). For the overall data of mineral concentrations, the regression analysis revealed weak to moderate relationships among EDTA whole blood and other blood specimens, suggesting the inferior use of this medium for determination of mineral profiles in dairy cows.

It is very important to justify why the diet composition, as a possible limitation of current research, was not analyzed. The study was not designed for detection the deficiency diseases of certain elements through analysis of minerals in the feeds to find the cause as the all essential minerals were supplemented in TMR, and all animals were clinically healthy as mentioned in materials and methods section. Moreover, each test animal ate a certain feed ration, which is, of course, the same for the different test specimen per animal. The specific feed ration is therefore irrelevant for the aim of the investigation. The most interesting is the different patterns of concentration of the various elements among the blood specimens.

## Conclusion

The concentrations of Cu, Zn, Fe, Mn, Ba, and Sr undergo physiological variations among dairy cows at different production stages. Moreover, the concentrations of essential and toxic elements varied among the different blood specimens. Therefore, during assessment of mineral profiles in dairy herds, caution should be taken to distinguish between the physiological changes, resultant from different lactation phases and deficiency diseases, as well as the variation among the type of blood specimens. Furthermore, the strong relationships between mineral concentrations in pool and individual samples were detected, recommending the use of pool sample measurements for the assessment of mineral status during herd supervision, instead of individual ones. In general, EDTA plasma may be more suitable for measurements of Ca, Mg, P, and S. It seems that EDTA plasma and heparinized plasma are suited for the estimation of Se and Fe, respectively.

## Data availability statement

The original contributions presented in the study are included in the article/Supplementary material, further inquiries can be directed to the corresponding author.

## Ethics statement

All animals were housed and cared according to the German Animal Welfare Act (S. 1206, 13137833–3/18.05.2006). The present study did not involve laboratory animals and only included blood samples. The blood sampling procedures reported herein were conducted according to Directive 2010/63/ EU and the regulations of the Institutional Animal Care and Use Committee, Free University of Berlin that follow the standard rules and regulations of OIE for use of animals in research purposes. Written informed consent was obtained from the owners for the participation of their animals in this study.

## Author contributions

HH: conceptualization, data curation, investigation, writing—original draft, writing—review, and editing. A-EM: data curation, formal analysis, writing—review, and editing. RS: conceptualization, data curation, investigation, writing—review, and editing. All authors have read and approved the manuscript.

## Conflict of interest

Author A-EM was employed by Vet Med Labor GmbH, IDEXX Laboratories. The remaining authors declare that the research was conducted in the absence of any commercial or financial relationships that could be construed as a potential conflict of interest.

## Publisher's note

All claims expressed in this article are solely those of the authors and do not necessarily represent those of their affiliated organizations, or those of the publisher, the editors and the reviewers. Any product that may be evaluated in this article, or claim that may be made by its manufacturer, is not guaranteed or endorsed by the publisher.

## Abbreviations

Cu: copper
Zn: zinc
Se: selenium
Mn: manganese
Ba: barium
Sr: strontium
Ca: calcium
Mg: magnesium
P: total phosphorous
S: sulfur
Co: cobalt
Si: silicon
Li: lithium
Ni: nickel
Tl: thallium
B: boron
Al: aluminum
U: uranium
As: arsenic
EDTA: ethylene diamine tetraacetic acid
TMR: totally mixed ration
ICP-OES: inductively coupled plasma–optical emission spectrometry
ICP-MS: inductively coupled plasma–mass spectrometry.
