# Supplementary material for: Comparative evaluation of mineral profiles in different blood specimens of dairy cows at different production phases
Source: Front Vet Sci. 2022 Oct 18;9:905249. doi: 10.3389/fvets.2022.905249 (PMC9622953; doi:10.3389/fvets.2022.905249)
Supplement: Supplementary file 1 [file Table_1.DOCX]

**Ingredients and chemical composition of the close-up and high lactation diet.**

| Feed ingredient or nutrient composition  (DM basis) | Close-up diet | High lactation diet |
| --- | --- | --- |
| Ingredients |  |  |
| Corn silage (%) | 37,5 | 36,5 |
| Grass silage (%) | 40,2 | 21,6 |
| Concentrate mineral mixture (%) | 22,3 | 41,9 |
|  |  |  |
| Nutrient composition |  |  |
| DM (%) | 35,7 | 41,5 |
| NEL (MJ/kg) | 6,3 | 6,9 |
| CP (%) | 15,0 | 162 |
| Ether extract (%) | 3,3 | 3,0 |
| aNDFom (%) | 43,3 | 37,2 |
| ADFom (%) | 24,6 | 21,3 |
| ADL (%) | 4,1 | 3,6 |
| Starch (%) | 14,1 | 25,6 |
| Sugar (%) | 3,1 | 3,8 |
| NFC (%) | 30,4 | 38,0 |
| Ash (%) | 8,0 | 6,6 |
| Ca (g/kg) | 6,0 | 6,7 |
| P (g/kg) | 4,3 | 3,8 |
| Mg (g/kg) | 3,2 | 2,5 |
| Na (g/kg) | 2,1 | 2,4 |
| K (g/kg) | 13,4 | 13,2 |
| Cl (g/kg) | 6,9 | 4,4 |
| S (g/kg) | 2,1 | 2,1 |
| DCAD (mEq/kg) | 109 | 193 |
| Fe (mg/kg) | 292 | 180 |
| Cu (mg/kg) | 16 | 14 |
| Zn (mg/kg) | 104 | 84 |
| Mn (mg/kg) | 80 | 66 |
| Se (mg/kg) | 0,49 | 0,28 |
| Co (mg/kg) | 0,40 | 0,31 |

DM dry matter; NEL energy concentration, CP crude protein, NDF neutral detergent fiber, ADF acid detergent fiber, ADL acid detergent lignin, NFC non fiber carbohydrates (calculated as 100-CP-ether extract –NDF-ash), DCAD = [(Na % of DM/0.023) + (K % of DM/0.039)] – [(S % of DM/0.016) + (Cl % of DM/0.0355)]
