# Supplementary material for: Comparative evaluation of mineral profiles in different blood specimens of dairy cows at different production phases
Source: Front Vet Sci. 2022 Oct 18;9:905249. doi: 10.3389/fvets.2022.905249 (PMC9622953; doi:10.3389/fvets.2022.905249)
Supplement: Supplementary file 2 [file Table_2.DOCX]

**Table 1: Variation coefficients (VC %) for analytical assys by Inductively Coupled Plasma - Mass Spectrometry (ICP-MS)**

| ICP MS | Element | Se 78 | Co59 | Ni 60 | As 75 | Li 7 | Tl 205 | U 238 |
| --- | --- | --- | --- | --- | --- | --- | --- | --- |
| Serum | Interassay (VC %) | 5,3 | 3,9 | 2,9 | 4,6 | 8,8 | 4,8 | 3,7 |
|  | Intraassay (VC %) | 3,8 | 0,8 | 2,3 | 2,2 | 3,9 | 2,7 | 2,3 |
| Plasma | Interassay (VC %) | 3,8 | 4 | 2 | 5 | 9,7 | 2,4 | 4,1 |
|  | Intraassay (VC %) | 4,1 | 1,8 | 2 | 2,2 | 6,3 | 2,5 | 2,8 |
| EDTA | Interassay (VC%) | 5,7 | 4,8 | 5,4 | 5,3 | 10 | 3,47 | 4,4 |
|  | Intraassay (VC %) | 3,2 | 1,9 | 1,8 | 2,3 | 5,7 | 2,8 | 3,4 |

**Table 2: Variation coefficients (VC %) for analytical assays by Inductively Coupled Plasma - Optical Emission Spectrometry (ICP-OES)**

| ICP-OES | Element | Cu 324,754 | Zn 202,548 | Mn 257,610 | | S 181,972 | P185,878 | Fe 238,204 | Ba 455,403 |
| --- | --- | --- | --- | --- | --- | --- | --- | --- | --- |
| Serum | Interassay (VC %) | 3,6 | 8,8 | 17,7 | | 6,8 | 13,2 | 4,7 | 6,5 |
|  | Intraassay (VC %) | 0,6 | 0,8 | 0,8 | | 0,5 | 0,7 | 3,5 | 0,6 |
| Plasma | Interassay (VC %) | 9,1 | 4,8 | 9 | | 8,1 | 10,7 | 1105 | 8,7 |
|  | Intraassay (VC %) | 5,2 | 4,7 | 4,2 | | 4,9 | 5,1 | 4,9 | 5,1 |
| EDTA | Interassay (VC %) | 8,5 | 5,2 | 8,7 | | 12 | 9,7 | 12,7 | 7,4 |
|  | Intraassay (VC %) | 3,7 | 6,7 | 3,3 | | 3,2 | 3,2 | 1,5 | 1,7 |
|  |  |  |  |  |  | |  |  |  |
| ICP-OES | Element | Sr 407,771 | Si 251,611 | Ca 315,887 | Al 167,017 | | B 249,772 | Mg 279,553 |  |
| Serum | Interassay (VC %) | 6 | 8,8 | 6,2 | 16,1 | | 10,3 | 4,7 |  |
|  | Intraassay (VC %) | 0,7 | 0,6 | 0,6 | 1,7 | | 4 | 1 |  |
| Plasma | Interassay (VC %) | 10,4 | 10,9 | 6,1 | 19,2 | | - | 6 |  |
|  | Intraassay (VC %) | 5,2 | 4,5 | 3,3 | 8,1 | | - | 4,1 |  |
| EDTA | Interassay (VC %) | 13,8 | 11,3 | 7,7 | - | | - | 9,2 |  |
|  | Intraassay (VC %) | 1,6 | 6 | 1,8 | - | | - | 1,8 |  |
