# Supplementary material for: Comparative evaluation of mineral profiles in different blood specimens of dairy cows at different production phases
Source: Front Vet Sci. 2022 Oct 18;9:905249. doi: 10.3389/fvets.2022.905249 (PMC9622953; doi:10.3389/fvets.2022.905249)
Supplement: Supplementary file 3 [file Table_3.DOCX]

**Limit of detection (LOD) and limit of quantification (LOQ) using Inductively Coupled Plasma - Mass Spectrometry (ICP-MS)**

| **Element** | **Unit** | **LOD** | **LOQ** |
| --- | --- | --- | --- |
| **Li 7** | **µg/L** | **0,0123** | **0,0410** |
| **Co59** | **µg/L** | **2,0494E-06** | **6,831E-06** |
| **Ni60** | **µg/L** | **1,6107E-05** | **5,369E-05** |
| **As75** | **µg/L** | **1,6062E-05** | **5,354E-05** |
| **Se78** | **µg/L** | **9,0938E-06** | **3,031E-05** |
| **Tl205** | **µg/L** | **0,0001** | **0,0004** |
| **Li 7** | **µg/L** | **0,0123** | **0,0410** |
| **U238** | **µg/L** | **0,0001** | **0,0005** |

**Limit of detection (LOD) and limit of quantification (LOQ) using Inductively Coupled Plasma - Optical Emission Spectrometry (ICP-OES)**

| **Element /nm** | **Unit** | **LOD** | **LOQ** |
| --- | --- | --- | --- |
| **Cu 324.754** | **mg/L** | **0,001** | **0,003** |
| **Zn 202.548** | **µg/L** | **4,499** | **14,998** |
| **Mn 257.610** | **µg/L** | **0,063** | **0,209** |
| **S 181.972** | **mg/L** | **0,019** | **0,063** |
| **P 185.878** | **mg/L** | **0,016** | **0,052** |
| **Fe 238.204** | **µg/L** | **0,967** | **3,222** |
| **Ba 455.403** | **µg/L** | **0,017** | **0,055** |
| **Sr 407.771** | **µg/L** | **0,018** | **0,059** |
| **Si 251.611** | **mg/L** | **0,001** | **0,004** |
| **Ca 315.887** | **mg/L** | **0,006** | **0,019** |
| **Al 167.019** | **µg/L** | **1,744** | **5,814** |
| **B 249.772** | **µg/L** | **1,937** | **6,458** |
| **Mg 279.553** | **mg/L** | **0,001** | **0,002** |
